# Methodological Challenges in Health Research during and after math of COVID-19 Pandemic Era

**DOI:** 10.31729/jnma.9127

**Published:** 2025-06-30

**Authors:** Srijana Bhattarai, Salabh Shah, Bhanu Bhakta Bashyal, Chandra Bahadur Sunar, Kaushalya Shrestha

**Affiliations:** 1 Modern Technical College of Health Science, Pokhara University, Sanepa Lalitpur, Nepal; 2 Abt Associates, Birendranagar, Surkhet, Nepal; 3 Provincial directorate, Birendranagar, Surkhet, Nepal; 4 Health Management Information System Section, Department of Health Services, Teku; 5 Tribhuwan University Teaching Hospital, Maharajgunj, Nepal

**Keywords:** *COWD-29*, *health research*, *research design*

## Abstract

The global pandemic of COVID-19 has had an impact on health, social and economic sectors. Health research is not exempt from the current crisis and is facing many challenges. The main challenges while conducting health research in the COVID-19 context include methodological challenges faced during data collection', 'difficulty in identifying non-verbal cues', 'time constraints' and 'ethical issues. Responding to the crisis, quantitative and qualitative research projects need some modifications to address the challenges of conducting research in the COVID-19 pandemic context. This commentary highlights the major challenges encountered while conducting research during the COVID-19 pandemic and provides ways quality research can be conducted in pandemic or other situations where physical distancing needs to be maintained.

## INTRODUCTION

The global pandemic of the COVID-19 has affected every aspect of human lives. Public health and social measures such as quarantine, isolation, physical distancing, wearing masks, hand washing were used effectively to control the pandemic.^1,2^ In these exceptional circumstances of COVID-19 pandemic, health researchers across the globe faced difficulties to carry out their research projects. The pandemic era demanded ordinary research methods to be adapted complying with COVID-19 safety protocols being considerate to both qualities of work and safety of the participants and researchers. Researchers encountered variety of challenges in their quantitative and qualitative research project amid the first wave of COVID-19 pandemic and it's after math. The objective of this viewpoint is to highlight the major challenges encountered while conducting health research during the COVID-19 pandemic and to propose adaptive strategies for ensuring methodological rigor, ethical integrity, and inclusivity in such crisis contexts.

## CHALLENGES

**1. Sampling and data collection method**

Quantitative research demands a good sample size following probability sampling to claim the results to be generalizable to the population and this is also a primary interest of quantitative researchers.^3,4^ Due to pandemic a balanced judgment of risk and benefit analysis needed to be employed before planning for the usual quantitative sampling methods like cluster sampling, population proportionate sampling because public health measures can help break transmission chain but are not bullet interventions like vaccination. The situation is no different to the qualitative researchers since they are interested more in observation of the ground realities, life and living missing the opportunity of field based research with use of methods like face-to-face interviews and discussions, and participant observation. The cases of different COVID variant over the time period is rising again which might put the researchers in the same situation.

In an already resource constrained setting, alternative methods like booming of electronic methods like online surveys, telephone interviews that are being advocated and practiced globally for data collection are challenging as the equity divide of technology comes into play.^5^ The technology divide affects research globally by limiting participation, data quality, and representation in low-resource or digitally disconnected regions.^5^ For population based online surveys, access of the people to technology along with computer literacy are additional challenges that one will face besides remarkable non-response rates. Similar is the situation for the telephone interviews where the access to proper network and audibility of voices can compromise the quality of data being which is challenging to qualitative researchers.

An effective adaptation technique is to use blended data collection methods, such as combining telephone or online interviews with support from local community health workers, to overcome challenges related to limited technological access and digital literacy.^2^

**2. Connectivity with participants and duration of data collection**

The struggle of the qualitative researchers to be able to get the connectivity built with their participants through virtual platforms is more than in a physical setting due to the restricted movements and demand of physical distancing during pandemic. While the online surveys risk the complete detachment of the quantitative researchers from their study participants. The data collection during quantitative interviews consume 20-30 minutes on an average from the participants during in-person interviews and holding participants over telephone for this period is very time consuming and annoying to respondents for giving their response.^4^ Managing such situation was very challenging for the researcher. The unpredictable rise of new variant of COVID again raise a question regarding the methodological challenge to be addressed by the researcher so that such shortcomings can be managed effectively in further time ahead.

The potential solution to the challenges is to build rapport with participant's in advance through preinterview phone calls or messages and to use shorter, well-structured interview guides specifically designed for remote communication, making the process more efficient and participant-friendly.^2^

**3. Difficulty in identifying non-verbal cues**

Identification of non-verbal cues such as gestures, body language of participants has great importance in qualitative research.^6^ It is difficult to identify these nonverbal cues during telephone and online qualitative interviews. Though non-verbal cues like pause, tone of voice can be noted in telephone and additionally facial expression noticed in online platforms using video facility. But many subtle cues like body posture, gestures, or micro-expressions are often missed or distorted due to poor connectivity, limited visibility, or repetition, affecting the natural flow and authenticity of responses. The challenge to the qualitative researchers is to make meaningful interpretations of the data that they have collected without these non-verbal cues.

An effective adaptation technique for this challenges is to conduct interviews using video conferencing platforms whenever possible, allowing researchers to observe facial expressions and some body language, while also training themselves to pay closer attention to vocal cues such as tone, pauses, and hesitations for richer interpretation.^6^

**4. Ethical issues**

The primary responsibility of the researcher is to protect the respondents from possible risks and harmful situations. In the widespread pandemic situation, the researcher may be the carrier for transmission of the virus since they get exposed to a large number of people throughout the day. The ethical review board will prioritize safeguarding participants from disease transmission risks just as much as the researcher, in addition to upholding routine ethical standards.^5^

With the promotion of the alternative strategy for data collection, for online surveys there is challenge of ensuring whether the participants understood the information sheet, questions like did they even read before they participated in areas where people are less familiar with the research protocols and participation in research remain unanswered. Alternative methods to take consent from participants in the current situation of COVID-19 pandemic like- emailed consent, phone consent, video conferencing and e-consent.^7^ The challenge is to be able to grab the local language like in a physical setting where the researcher may have an option of having a standby translator where required.

Further to it, studies without the agenda of COVID-19 have faced challenges of getting timely ethical review on their protocols and even publications. Using verbal or electronic consent procedures supported by simplified information sheets in the local language, delivered through phone, video, or messaging platforms, along with comprehension checks to ensure informed participation while minimizing physical contact can adapt to the challenges encounterd.^5^

**5. Double burden of pandemic situation related to vulnerable population**

COVID-19 has had a significant socioeconomic effect leading to job losses, financial crises, and families which may grossly increase the respondent burden, particularly among marginalized populations. When these vulnerable participants take part in research it may be relatively more difficult, and emotionally stressful that may lead to data incompleteness and even non-responses in one hand. At the same time the voice of the vulnerable may not be reached with these alternative strategies developed during COVID-19 pandemic who indeed are the ones at higher social disruption. This technology divide makes inferences, in the studies done during the pandemic, challenged in terms of generalizability. Engaging trusted community intermediaries to reach vulnerable populations and use context-appropriate, low-tech methods such as phone interviews or paper-based tools to reduce participant burden and ensure their voices are included in the research could be the potential solution for the given challenges^5^

## WAY FORWARD

COVID-19 has not stopped the researchers with the adaptation of alternative strategies and fintech adjusted for the pandemic scenario. Major challenges being faced is the technological divide. The researchers need to ensure data quality and inclusivity to have least compromise with the meaningful interpretations, inferences and conclusions they come with in the research done during the pandemic. To improve data collection in diverse settings, researchers should employ mixed-mode data collection methods, combining online, phone, and safe in-person approaches. Simplifying tools and enhancing digital literacy among participants can help ensure broader access and understanding. Developing flexible, ethically sound protocols that can adapt to emergencies is also essential. Collaborating with local stakeholders enables more effective outreach to vulnerable groups. Further research is needed to evaluate how remote methods affect data quality, representation, and participant well-being, and to establish standardized ethical guidelines for conducting research during pandemics.
